# The Prolonged Hospital Stays at Nigerian Teaching Hospitals: Roles of Medical Social Workers

**DOI:** 10.4314/ejhs.v34i5.11

**Published:** 2024-09

**Authors:** Oluwagbemiga Oyinlola, Raimi Olalekan Adeleke, Abimbola Afolabi

**Affiliations:** 1 McGill University, School of Social Work, Montreal, Canada; Medical Social Services Department, University College Hospital, Ibadan; 2 Medical Social Services Department, University College Hospital, Ibadan; Department of Adult Education, University of Ibadan; 3 Department of Social Work, University of Ibadan

**Keywords:** Case study, prolonged hospital admission, medical social workers, Nigeria

## Abstract

Prolonged hospital stays in Nigerian teaching hospitals pose a significant challenge to patient care and hospital management, exacerbated by socio-economic and systemic factors. This case study report looked at the multifaceted role of medical social workers in addressing these challenges, focusing on their efforts in providing psychosocial support, coordinating care, and advocating for patients within a strained healthcare system. This case-study highlights the impact of resource constraints and inadequate hospital practices on patient outcomes, emphasizing the psychological toll on patients and their families. It underscores the critical role of medical social workers as they navigate complex healthcare landscapes to mitigate the adverse effects of extended hospitalizations. This calls for a comprehensive approach to address these systemic issues, including policy reforms, increased healthcare funding, and strategic improvements in hospital administration. Hence the urgency of systemic change to ensure a more resilient and compassionate healthcare environment for all Nigerians.

## Introduction

The issue of prolonged hospital stays is a complex problem with significant implications for patient care and hospital management. In Nigerian teaching hospitals, this challenge is exacerbated by various socio-economic and healthcare system factors. Medical social workers play a vital role in addressing these complexities, as demonstrated by studies on their interventions within the broader healthcare environment in Nigeria(1,2). While financial constraints often lead to discharge against medical advice (DAMA), which can contribute to prolonged stays, medical social workers focus on education and legal facilitation to prevent litigation. This letter aims to illuminate the impact of extended stays in teaching hospitals and the urgent need for systemic changes to mitigate the burdens on patients, healthcare professionals, and social workers alike.

Nigerian teaching hospitals are crucial for medical education and specialized healthcare services(3). However, they face systemic challenges, including infrastructural inadequacies, policy gaps, and limited resources(3-6). Overcrowding and systemic inefficiencies often result in prolonged hospital stays, which limit the hospitals' ability to admit new patients and increase the risk of hospital-acquired infections.

Medical social workers encounter several challenges, such as a lack of legal backing and inadequate recognition, which hinder their effectiveness in public health facilities(10,11). Limited availability of nursing home facilities and cultural factors complicate the transition of older adults from hospitals to nursing homes. Social workers, who are tasked with addressing the psychosocial needs of patients and their families, struggle to provide effective support, coordinate discharge planning, and manage scarce resources. This leads to heightened stress and burnout among social work professionals, compromised patient care, and strained patient-family dynamics, further complicating the social workers' mission to facilitate holistic healing and discharge planning(6,12-14). The psychological and emotional toll of extended hospitalization on patients and their families is significant, with isolation, anxiety, and depression being common among patients(10,11,15). The support of clinical social workers is crucial in these situations, but overwhelming caseloads and resource limitations often impede their ability to provide necessary psychosocial support, adversely affecting patient mental health and overall well-being.

Addressing this issue requires a comprehensive strategy that includes policy changes, increased financial resources for healthcare, and improved hospital administration and patient flow management. Enhancing healthcare infrastructure, streamlining patient management systems, and providing better support and training for clinical social workers are essential components of this approach.

## Cases

### Case Example 1: Impact of resource constraints

Mrs. Adebayo, a 58-year-old widow, was admitted with complications related to diabetes. Due to limited availability of specialized medical equipment and frequent shortages of essential medications, her treatment was delayed, extending her hospital stay. This heightened her risk of further health complications and imposed a financial strain on her family. Medical social workers provided crucial psychosocial support, helping Mrs. Adebayo and her family navigate the healthcare system and connect with community resources. They advocated for her access to necessary treatments, coordinated her care, and provided emotional support to alleviate the stress of her prolonged hospitalization and financial burden.

### Case Example 2: Strain on mental health

Mr. Chinedu, a 35-year-old teacher, experienced a prolonged stay following a routine surgery due to post-operative infections linked to inadequate hospital hygiene practices. This extended hospitalization affected his mental health, leading to depression and anxiety, which complicated his recovery. Medical social workers assessed his emotional and psychological well-being, facilitated access to mental health services, and supported his family in understanding and coping with the situation. They aimed to ensure a comprehensive and compassionate approach to Mr. Chinedu's overall care.

The challenges associated with prolonged hospital stays in Nigerian teaching hospitals, as illustrated by the cases of Mrs. Adebayo and Mr. Chinedu, reflect deeper systemic issues within the healthcare sector. Medical social workers must proactively address these challenges through advocacy, holistic care, and policy reform. To improve healthcare delivery and outcomes, concerted efforts and systemic change are necessary. Immediate action is required from hospital administrators, government health officials, and the broader health policy community to address these inefficiencies. Collaborative efforts are needed to develop a resilient, responsive, and compassionate healthcare system.

## Discussion

This case report highlights critical challenges associated with prolonged hospital stays in Nigerian teaching hospitals, with a focus on the essential yet undervalued role of medical social workers. It examines the systemic deficiencies within the healthcare system that contribute to extended hospitalizations and critiques the limitations of the medical social work profession in addressing these issues effectively. One of the primary problems identified is the lack of resources in Nigerian hospitals, exemplified by the case of Mrs. Adebayo. Her delayed treatment due to shortages of equipment and medications reflects deeper structural inequities and chronic underfunding in the healthcare system. The inability to provide timely medical care stems not only from operational inefficiencies but also from political neglect and poor prioritization of healthcare funding(9,11). These factors exacerbate inequities in patient care and place immense stress on medical social workers, who are expected to advocate for and support patients in an environment that lacks the necessary resources. This creates a paradox where social workers are tasked with helping patients but are disempowered by the very system in which they work.

Medical social workers, while attempting to provide psychosocial support, are overburdened and under-supported, making it difficult for them to address the comprehensive mental health needs of patients. This cycle of neglect and strain illustrates the broader systemic failure to integrate psychosocial care into the patient's overall treatment plan(8). The report also emphasizes the undervaluation of medical social workers. These professionals face numerous challenges, such as high caseloads, lack of legal backing, and limited recognition within the healthcare system. Their contributions to patient care, especially in addressing emotional and social needs, are often overlooked in a system that prioritizes biomedical interventions over holistic care. This marginalization further limits their ability to provide effective support, creating a gap in patient care that prolongs hospitalizations and worsens patient outcomes.

From a policy standpoint, the case report calls for urgent systemic reforms to address these root causes. Simply increasing hospital funding or addressing overcrowding will not resolve the underlying issues. A more comprehensive approach is needed, one that recognizes the social determinants of health and integrates psychosocial care into the healthcare model. Medical social workers must be empowered with better resources, legal recognition, and enhanced training to meet the complex needs of patients with prolonged hospital stays. This requires not only changes within the healthcare system but also a broader societal shift in the value placed on psychosocial care Without systemic changes that address resource allocation, healthcare management, and the psychosocial needs of patients, the challenges of prolonged hospital stays will persist. Medical social workers need greater support and recognition to fulfill their roles effectively, making this a call to action for healthcare administrators and policymakers to rethink how care is delivered.
